# Biomarker-integrated machine learning models for predicting treatment-related bowel dysfunction after rectal cancer surgery: a multicenter development and external validation study

**DOI:** 10.3389/fmed.2026.1907311

**Published:** 2026-08-10

**Authors:** Qiyuan Liu, Yunxin Liu, Yuxiang Huang, Yuchen Fu, Minglian Ouyang, Tao Liu, Jiahao Rao, Zihao Xia, Xinxin Liu, Zhixin Zeng, Junlin Zeng, Xiangfu Zeng, Shufeng Zhao

**Affiliations:** 1 Department of Gastrointestinal Surgery, First Affiliated Hospital of Gannan Medical University, Ganzhou, China; 2 School of Information and Software Engineering, East China Jiaotong University, Nanchang, China; 3 Department of Emergency Medicine, Anyang Regional Hospital, Puyang, China; 4 School of Nursing, Gannan Medical University, Ganzhou, China; 5 First School of Clinical Medicine, Gannan Medical University, Ganzhou, China; 6 Nanchang University, Nanchang, China

**Keywords:** biomarker-driven prediction, clinical biomarkers, external validation, low anterior resection syndrome, machine learning, multicenter study, personalized cancer therapy, rectal cancer

## Abstract

**Background:**

Low anterior resection syndrome (LARS) is a prevalent treatment-related complication following sphincter-preserving surgery for rectal cancer that severely impairs survivors’ quality of life. In the era of biomarker-driven personalized cancer therapy, reliable tools integrating clinicopathological biomarkers for individualized risk stratification remain lacking. Machine learning (ML) approaches offer the potential to integrate heterogeneous biomarkers—including nutritional, inflammatory, and treatment-related indicators—for precision prediction, yet their comparative performance against conventional nomograms in LARS prediction has not been rigorously evaluated in multicenter settings.

**Methods:**

This multicenter retrospective cohort included 906 patients undergoing laparoscopic low anterior resection for rectal adenocarcinoma, with 506 in the training cohort and 400 from four hospitals for external validation. Major LARS was defined as a 6-month LARS score ≥ 21. LASSO selected features from 24 candidate variables. Five machine-learning models and a logistic regression nomogram were evaluated using AUC, calibration, decision curve analysis, and SHAP interpretation.

**Results:**

The incidence of major LARS was 48.0% (243/506) in the training cohort and 52.2% (209/400) in the validation cohort. LASSO regression selected 14 predictive biomarkers, including preoperative serum albumin, neoadjuvant chemotherapy, and preoperative radiotherapy. In external validation, the random forest model achieved the highest AUC of 0.903 (sensitivity 0.871, specificity 0.796), followed by the logistic regression nomogram (AUC 0.888). Multivariate analysis identified tumor–anal verge distance (OR = 0.733, 95% CI 0.679–0.792, *p* < 0.001), stoma reversal time (OR = 1.240, *p* < 0.001), anastomotic leakage (OR = 7.027, *p* = 0.004), and diabetes mellitus (OR = 2.793, *p* = 0.006) as independent risk factors. SHAP analysis confirmed tumor–anal verge distance as the dominant predictor, with preoperative albumin ranking fifth in feature importance, demonstrating the capacity of ML to extract predictive signal from clinically relevant but statistically marginal biomarkers.

**Conclusion:**

The random forest model integrating clinicopathological biomarkers and treatment-related variables demonstrated robust predictive performance for postoperative or post-treatment risk stratification of treatment-related bowel dysfunction (LARS). The model is intended to support postoperative monitoring, survivorship counseling, and rehabilitation planning rather than purely preoperative treatment selection. These biomarker-driven prediction tools can facilitate individualized functional-risk assessment in rectal cancer patients undergoing multimodal therapy.

## 1 Introduction

Rectal cancer is one of the most common gastrointestinal malignancies, with over 730,000 new cases diagnosed annually worldwide (1). With advances in surgical techniques and multimodal treatment strategies, sphincter-preserving low anterior resection (LAR) has become the standard-of-care for mid-to-low rectal cancer, enabling avoidance of permanent colostomy (2). However, a substantial proportion of patients who undergo LAR subsequently develop low anterior resection syndrome (LARS), a constellation of distressing bowel symptoms including fecal incontinence, urgency, increased stool frequency, and clustering of defecation (2–4). The reported incidence of major LARS ranges from 30% to 76% across published series (5, 6), and this debilitating condition profoundly compromises patients’ health-related quality of life, social functioning, and psychological wellbeing (7, 8). More broadly, postoperative patient-reported quality of life and functional recovery are increasingly recognized as clinically important survivorship endpoints after cancer surgery (9).

In the current era of biomarker-driven cancer therapy, the identification of predictive biomarkers to guide personalized treatment decisions has become a central paradigm in oncology (10, 11). Neoadjuvant chemoradiotherapy (nCRT), while essential for local tumor control and pathological downstaging, exerts well-documented immunomodulatory effects on the tumor microenvironment and surrounding tissues, including the pelvic floor musculature, enteric nervous system, and intestinal mucosal immunity (10–13). Recent work across tumor types further emphasizes that tumor immune microenvironmental states can differ substantially according to host and tumor biological context, reinforcing the need to interpret treatment-related functional outcomes within a broader immune-tissue response framework (14). Radiation-induced fibrosis, mucosal inflammation, and pelvic neuropathy have been implicated as contributors to long-term bowel dysfunction (15–17). Nutritional biomarkers such as serum albumin have been recognized as surrogate indicators of systemic inflammatory status and immune competence, with hypoalbuminemia consistently associated with adverse surgical outcomes across cancer types (18–21). However, despite the clinical significance of these treatment-related and nutritional biomarkers, no systematic effort has been made to integrate them into a validated predictive framework for LARS (22–26).

Several clinicopathological risk factors have been associated with LARS, including tumor location relative to the anal verge, anastomotic complications, diverting stoma creation, and neoadjuvant therapy (5, 6, 27, 28). Existing prediction tools, such as the POLARS score (6) and various single-center nomograms (24, 25), have provided initial frameworks but are limited by their reliance on conventional logistic regression, which assumes linear relationships between predictors and outcomes and may fail to capture complex, nonlinear biomarker interactions. Machine learning (ML) approaches offer the capacity to model high-dimensional, nonlinear relationships without restrictive parametric assumptions (29, 30). Recent applications of ML in oncology have demonstrated promising performance in predicting treatment response, postoperative complications, and survival outcomes (29, 30). Notably, Qin et al. (22) developed a multicenter ML model for LARS prediction that achieved an AUC of 0.852 in external validation, while Chen et al. (23) applied ML techniques to a single-center LARS dataset. However, head-to-head comparisons between multiple ML algorithms and conventional nomograms with comprehensive external validation and model interpretability analyses remain scarce.

To address these gaps, the present study aimed to: (1) develop and externally validate multiple ML prediction models incorporating clinicopathological biomarkers—including nutritional indicators and treatment-related factors—for major LARS following LAR for rectal cancer; (2) compare the predictive performance of ML models against a conventional logistic regression–based nomogram; (3) identify independent risk factors through multivariate analysis; and (4) employ SHapley Additive exPlanations (SHAP) (31) to enhance model interpretability and elucidate the relative contributions of individual biomarkers. We hypothesized that biomarker-integrated ML models would provide superior predictive accuracy while offering enhanced capability for capturing nonlinear biomarker–outcome relationships, thereby supporting personalized risk stratification in rectal cancer patients undergoing multimodal therapy.

## 2 Materials and methods

### 2.1 Study design and patient population

This multicenter retrospective cohort study was conducted across five medical centers in Jiangxi Province, China. The training cohort was derived from the First Affiliated Hospital of Gannan Medical University (*n* = 506, October 2019–September 2025). The external validation cohort comprised patients from Ganzhou People’s Hospital (*n* = 302), Yudu People’s Hospital (*n* = 46), Nankang District People’s Hospital (*n* = 37), and Ruijin General Hospital (*n* = 15), covering October 2022 to September 2025 (total *n* = 400). The study was approved by the institutional review board and reported in accordance with the TRIPOD guidelines (32). Because source screening logs were not uniformly retrievable from all participating centers, excluded-patient counts by individual exclusion reason could not be reconstructed reliably; this limitation was considered when interpreting transportability.

### 2.2 Inclusion and exclusion criteria

Inclusion criteria: (1) pathological diagnosis of rectal adenocarcinoma; (2) underwent laparoscopic LAR or Dixon procedure; (3) complete medical records and follow-up. Exclusion criteria: (1) concurrent malignancies; (2) preoperative distant metastasis; (3) palliative resection or conversion to open surgery; (4) carcinoma *in situ*; (5) postoperative recurrence or metastasis; (6) unreversed or permanent stoma; (7) death or inability to cooperate; (8) missing key data.

### 2.3 Data collection and candidate biomarkers

Twenty-four candidate predictor variables were categorized into four biomarker domains. Demographic and comorbidity variables: age, sex, BMI, diabetes mellitus, hypertension. Nutritional biomarkers: preoperative serum albumin (g/L), postoperative serum albumin (g/L), and perioperative albumin change (Δalbumin) (18–21). Tumor characteristics: tumor–anal verge distance (cm), distal resection margin distance (cm), tumor diameter (cm), T stage, N stage, and TNM stage (AJCC 8th edition). Treatment and surgical factors: neoadjuvant chemotherapy, preoperative radiotherapy, adjuvant chemotherapy, postoperative radiotherapy, operation time, prophylactic stoma, stoma reversal time, anastomotic leakage, time to first flatus, and drainage duration.

### 2.4 Outcome definition

Major LARS was assessed at 6 months postoperatively using the validated LARS score (2, 3), which ranges from 0 to 42 points and classifies bowel dysfunction as no LARS, minor LARS, or major LARS. In this study, major LARS was defined as a total score ≥21 (33). The Chinese questionnaire was administered during outpatient follow-up or structured telephone follow-up by trained study personnel, and questionnaires with incomplete key items were excluded from the final analysis.

### 2.5 Statistical analysis

Feature selection was performed using LASSO penalized logistic regression (34) with 10-fold cross-validation. Six models were constructed: logistic regression (LR), random forest (RF) (35), XGBoost (36), LightGBM (37), support vector machine (SVM), and multilayer perceptron (MLP). Model performance was evaluated using AUC (38), calibration curves, decision curve analysis (DCA) (39), and Brier scores. SHAP (31) analysis was applied to the best-performing model. Analyses were performed using Python 3.12 and R 4.3. A two-sided *p* < 0.05 was considered significant (32, 40). All feature selection, preprocessing, scaling, hyperparameter tuning, and threshold selection were performed within the training cohort only, and the external validation cohort was kept untouched until final evaluation. Complete-case analysis was used for modeling; patients with missing key predictors or incomplete LARS questionnaires were excluded according to the predefined criteria. Hyperparameters for tree-based models, SVM, and MLP were selected by grid search within the training data using cross-validation. Class imbalance was examined by comparing event proportions in the training and validation cohorts.

## 3 Results

### 3.1 Patient characteristics

A total of 906 patients were included, comprising 506 in the training cohort and 400 in the validation cohort. The incidence of major LARS was 48.0% (243/506) and 52.2% (209/400), respectively (Table 1). The validation cohort had older patients (66.6 vs. 62.0 years, *p* < 0.001), higher rates of neoadjuvant chemotherapy (26.0% vs. 7.5%, *p* < 0.001), and preoperative radiotherapy (8.3% vs. 2.2%, *p* < 0.001).

**Table 1:** Baseline characteristics of the training and external validation cohorts.

Variable	Training (*n* = 506)	Validation (*n* = 400)	*p*-value
Age (years)	62.0 ± 10.1	66.6 ± 10.4	**<0.001**
BMI (kg/m^2^)	22.5 ± 3.0	22.4 ± 3.0	0.790
Preoperative albumin (g/L)	40.1 ± 3.7	38.5 ± 5.0	**<0.001**
Postoperative albumin (g/L)	34.8 ± 3.6	36.9 ± 4.0	**<0.001**
Operation time (min)	202.8 ± 65.4	247.4 ± 40.4	**<0.001**
Time to first flatus (days)	3.3 ± 1.3	3.8 ± 1.3	**<0.001**
Drainage duration (days)	9.7 ± 3.8	7.9 ± 1.8	**<0.001**
Tumor–anal distance (cm)	8.2 ± 3.4	7.8 ± 3.5	**0.020**
Distal margin distance (cm)	6.3 ± 3.1	5.8 ± 3.3	**0.005**
Tumor size (cm)	3.8 ± 1.7	3.8 ± 1.3	0.836
Stoma reversal time (months)	1.6 ± 2.4	2.4 ± 4.5	0.573
Male sex, *n* (%)	286 (56.5)	264 (66.0)	**0.005**
Diabetes mellitus, *n* (%)	52 (10.3)	33 (8.2)	0.355
Hypertension, *n* (%)	111 (21.9)	74 (18.5)	0.234
Prophylactic stoma, *n* (%)	160 (31.6)	127 (31.8)	1.000
Adjuvant chemotherapy, *n* (%)	305 (60.3)	202 (50.5)	**0.004**
Neoadjuvant chemotherapy, *n* (%)	38 (7.5)	104 (26.0)	**<0.001**
Preoperative radiotherapy, *n* (%)	11 (2.2)	33 (8.3)	**<0.001**
Postoperative radiotherapy, *n* (%)	20 (4.0)	37 (9.2)	**0.002**
Anastomotic leakage, *n* (%)	24 (4.7)	24 (6.0)	0.491
TNM stage I, *n* (%)	156 (30.8)	92 (23.0)	—
TNM stage II, *n* (%)	150 (29.6)	109 (27.3)	—
TNM stage III, *n* (%)	200 (39.5)	199 (49.8)	—
Major LARS, *n* (%)	243 (48.0)	209 (52.2)	0.222

Continuous variables: mean ± SD. Bold *p*-values indicate *p* < 0.05.

### 3.2 Univariate analysis

Univariate analysis (Table 2) identified tumor–anal verge distance (6.6 ± 2.7 vs. 9.7 ± 3.3 cm, *p* < 0.001), prophylactic stoma (48.1% vs. 16.3%, *p* < 0.001), anastomotic leakage (8.2% vs. 1.5%, *p* < 0.001), operation time (214.5 vs. 192.0 min, *p* < 0.001), and diabetes (13.6% vs. 7.2%, *p* = 0.019) as significant factors. Preoperative radiotherapy (4.5% vs. 0%, *p* = 0.001) and neoadjuvant chemotherapy (9.9% vs. 5.3%, *p* = 0.052) also showed associations. Preoperative albumin did not differ significantly between groups (40.1 ± 3.8 vs. 40.1 ± 3.7 g/L, *p* = 0.956).

**Table 2:** Univariate analysis of risk factors for major LARS in the training cohort.

Variable	LARS (*n* = 243)	Non-LARS (*n* = 263)	Test	*p*-value
Age (years)	61.1 ± 9.4	62.8 ± 10.7	U	**0.029**
BMI (kg/m^2^)	22.6 ± 3.1	22.4 ± 2.8	U	0.812
Preop albumin (g/L)	40.1 ± 3.8	40.1 ± 3.7	U	0.956
Postop albumin (g/L)	34.6 ± 3.6	35.0 ± 3.6	U	0.253
Operation time (min)	214.5 ± 64.9	192.0 ± 64.0	U	**<0.001**
Time to first flatus (days)	3.3 ± 1.3	3.3 ± 1.2	U	0.708
Drainage duration (days)	9.8 ± 4.1	9.7 ± 3.4	U	0.916
Tumor–anal distance (cm)	6.6 ± 2.7	9.7 ± 3.3	U	**<0.001**
Distal margin distance (cm)	4.8 ± 2.4	7.6 ± 3.1	U	**<0.001**
Tumor size (cm)	3.9 ± 1.7	3.8 ± 1.6	U	0.497
Stoma reversal time (months)	2.4 ± 2.8	0.8 ± 1.7	U	**<0.001**
Male sex, *n* (%)	143 (58.8)	143 (54.4)	*χ* ^2^	0.310
Diabetes, *n* (%)	33 (13.6)	19 (7.2)	*χ* ^2^	**0.019**
Hypertension, *n* (%)	45 (18.5)	66 (25.1)	*χ* ^2^	0.074
Prophylactic stoma, *n* (%)	117 (48.1)	43 (16.3)	*χ* ^2^	**<0.001**
Adjuvant chemo, *n* (%)	154 (63.4)	151 (57.4)	*χ* ^2^	0.171
Neoadjuvant chemo, *n* (%)	24 (9.9)	14 (5.3)	*χ* ^2^	0.052
Preop radiotherapy, *n* (%)	11 (4.5)	0 (0.0)	Fisher	**0.001**
Postop radiotherapy, *n* (%)	15 (6.2)	5 (1.9)	*χ* ^2^	**0.014**
Anastomotic leakage, *n* (%)	20 (8.2)	4 (1.5)	Fisher	**<0.001**

U, Mann–Whitney *U* test; *χ*^2^, chi-square test; Fisher, Fisher’s exact test. Bold: *p* < 0.05.

### 3.3 Feature selection

**Figure 1:**
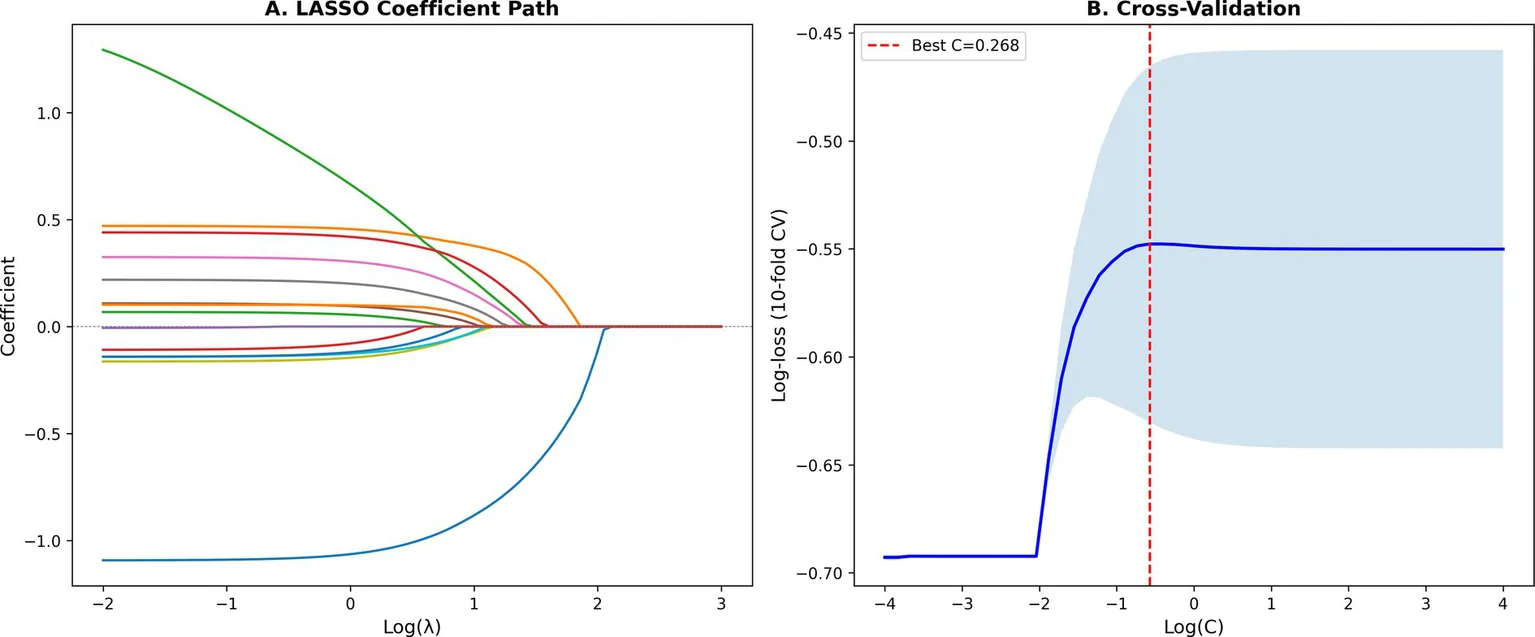
LASSO feature selection. **(A)** Coefficient path showing the shrinkage of predictor coefficients as the regularization parameter increases. **(B)** Ten-fold cross-validation curve for selecting the optimal regularization parameter.

LASSO regression selected 14 features (Figure 1): tumor–anal verge distance, stoma reversal time, preoperative radiotherapy, anastomotic leakage, N stage, T stage, diabetes, postoperative radiotherapy, age, hypertension, preoperative serum albumin, tumor size, drainage duration, and neoadjuvant chemotherapy. Notably, preoperative albumin was retained despite non-significance in univariate analysis, suggesting a contribution within the multivariate biomarker context.

### 3.4 Multivariate logistic regression

Four independent predictors were identified (Table 3): tumor–anal verge distance (OR = 0.733, 95% CI 0.679–0.792, *p* < 0.001), stoma reversal time (OR = 1.240, 95% CI 1.124–1.369, *p* < 0.001), anastomotic leakage (OR = 7.027, 95% CI 1.876–26.325, *p* = 0.004), and diabetes (OR = 2.793, 95% CI 1.352–5.766, *p* = 0.006). Preoperative albumin showed a protective trend (OR = 0.972, 95% CI 0.914–1.034, *p* = 0.370).

**Table 3:** Multivariate logistic regression analysis for major LARS.

Variable	OR	95% CI	*p*-value
Tumor–anal distance (per cm)	0.733	0.679–0.792	<0.001***
Stoma reversal time (per month)	1.240	1.124–1.369	<0.001***
Anastomotic leakage	7.027	1.876–26.325	0.004**
Diabetes mellitus	2.793	1.352–5.766	0.006**
Postoperative radiotherapy	2.534	0.755–8.505	0.132
Age (per year)	0.985	0.963–1.007	0.170
Hypertension	0.734	0.429–1.258	0.261
Preoperative albumin (per g/L)	0.972	0.914–1.034	0.370
T stage (per level)	1.116	0.875–1.424	0.377
Tumor size (per cm)	1.063	0.923–1.225	0.396
Neoadjuvant chemotherapy	1.373	0.576–3.273	0.475
Drainage duration (per day)	1.018	0.957–1.083	0.563
N stage (per level)	1.008	0.725–1.400	0.964

OR, odds ratio; CI, confidence interval. ***p < 0.001; **p < 0.01. Preoperative radiotherapy excluded due to complete separation.

### 3.5 Prediction model performance

**Figure 2:**
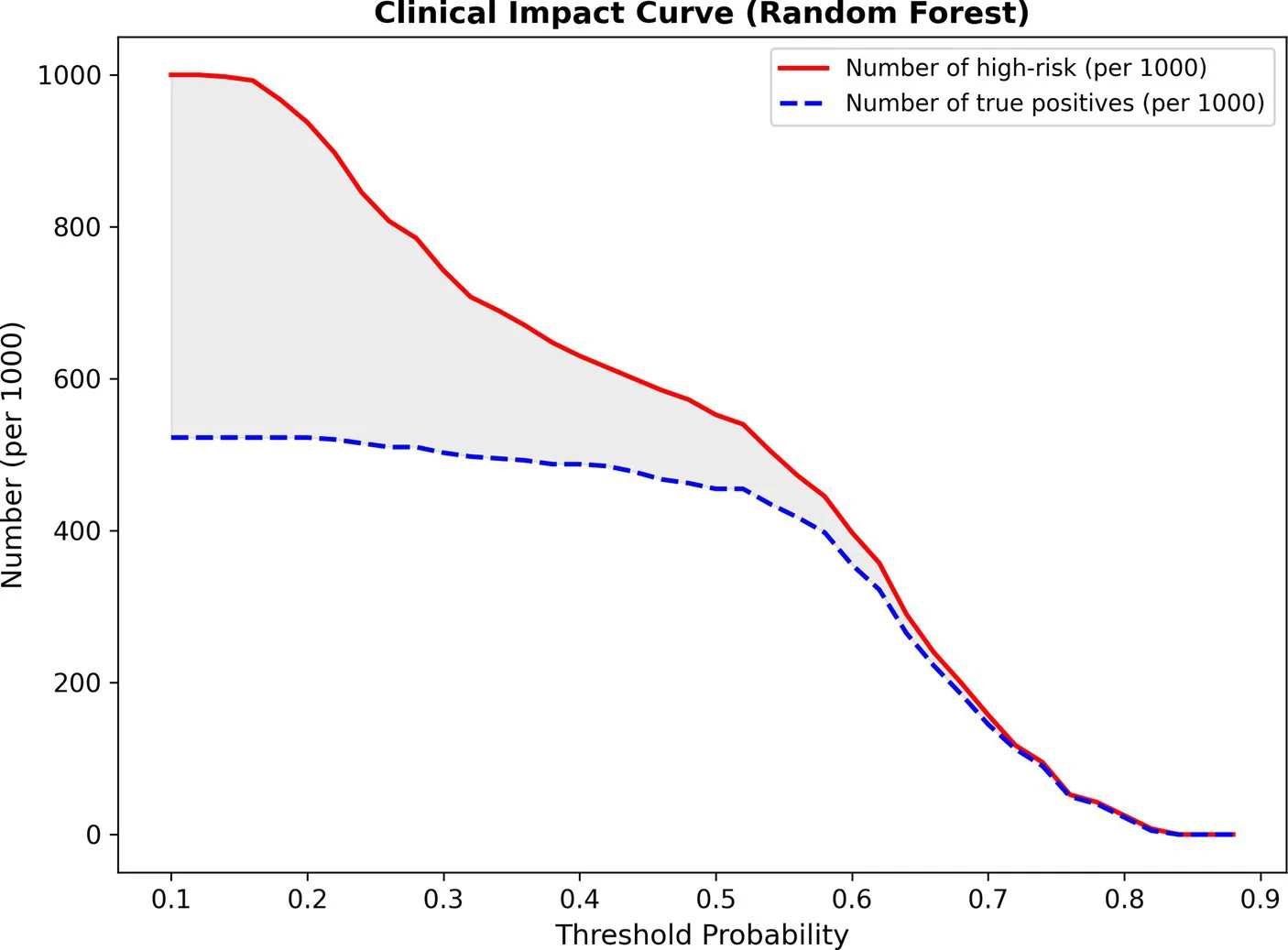
Receiver operating characteristic (ROC) curves of six prediction models in the training cohort (left) and external validation cohort (right). Model labels correspond to the order used in Table 4.

**Figure 3:**
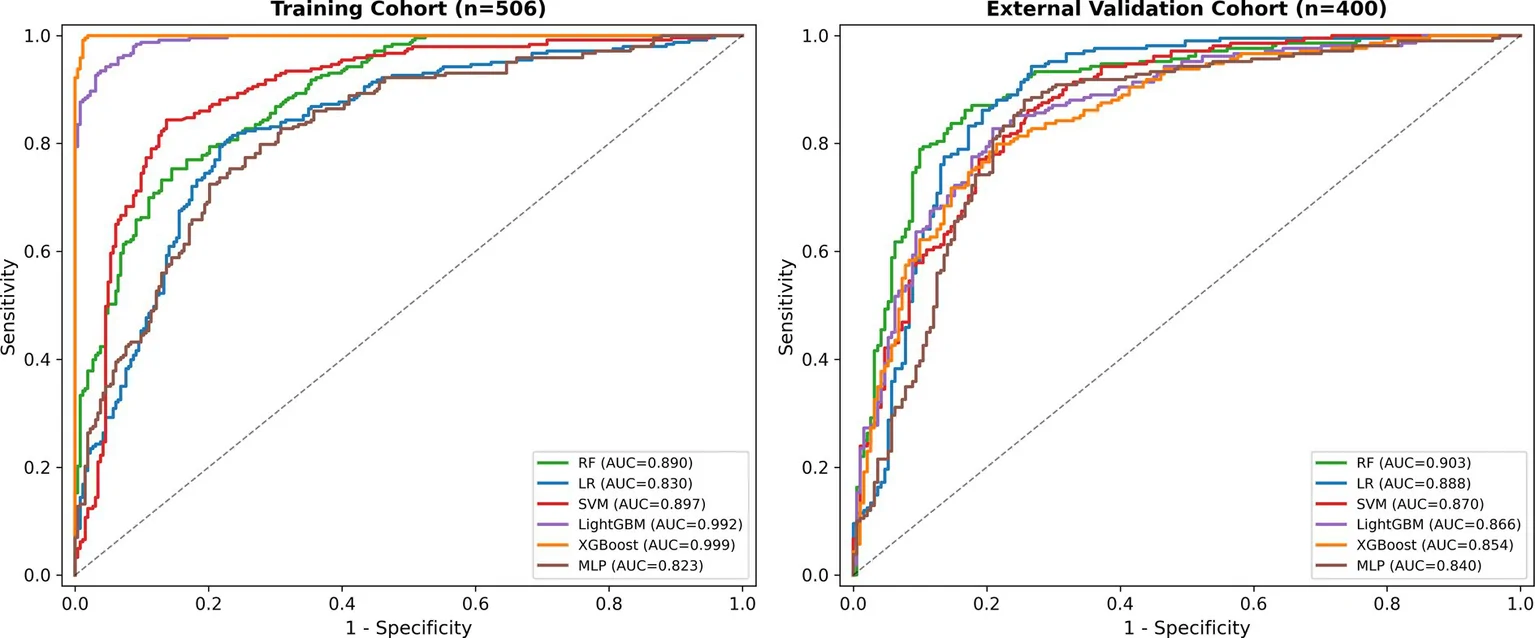
Calibration curves of six prediction models in the training cohort (left) and external validation cohort (right). Model labels correspond to the order used in Table 4.

**Figure 4:**
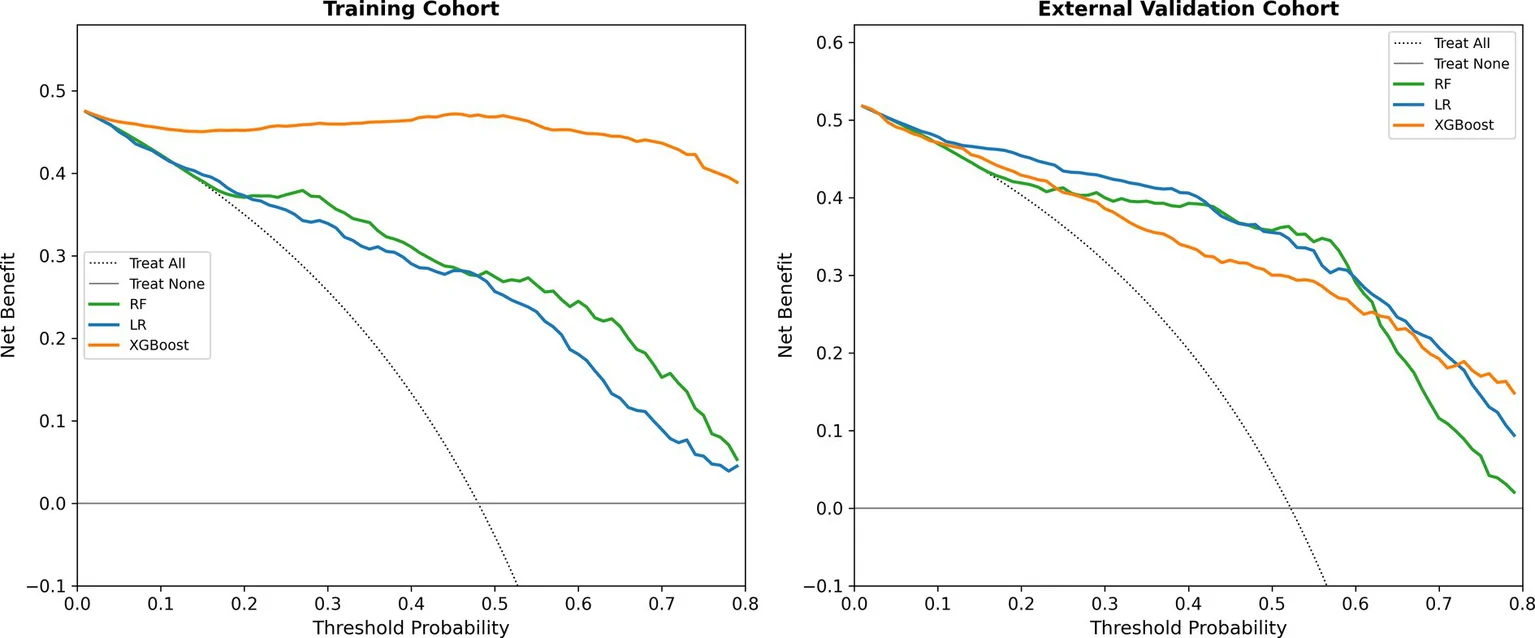
Decision curve analysis (DCA) of the top three prediction models in the training cohort (left) and external validation cohort (right).

**Figure 5:**
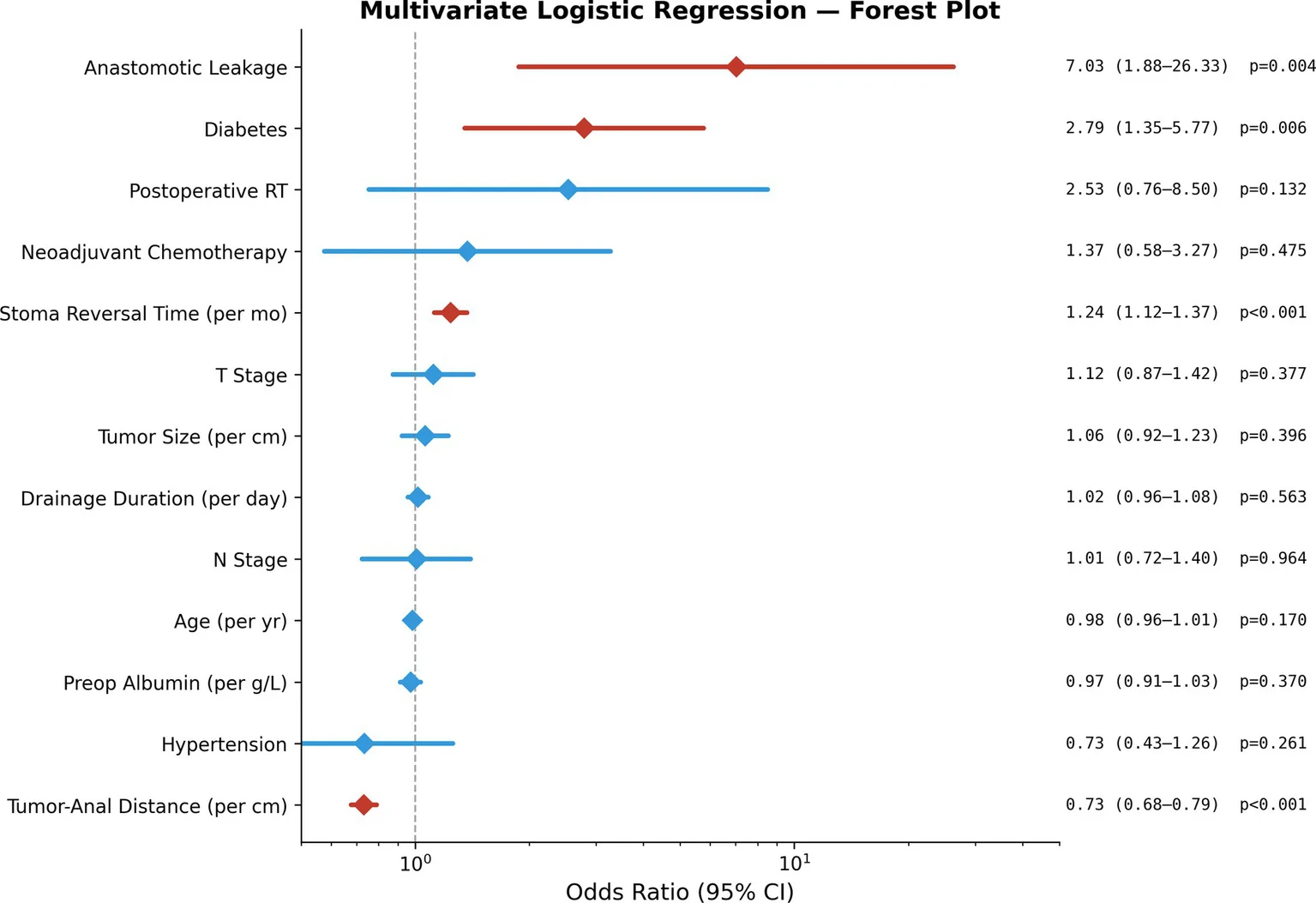
Clinical impact curve of the random forest model in the external validation cohort.

**Figure 6:**
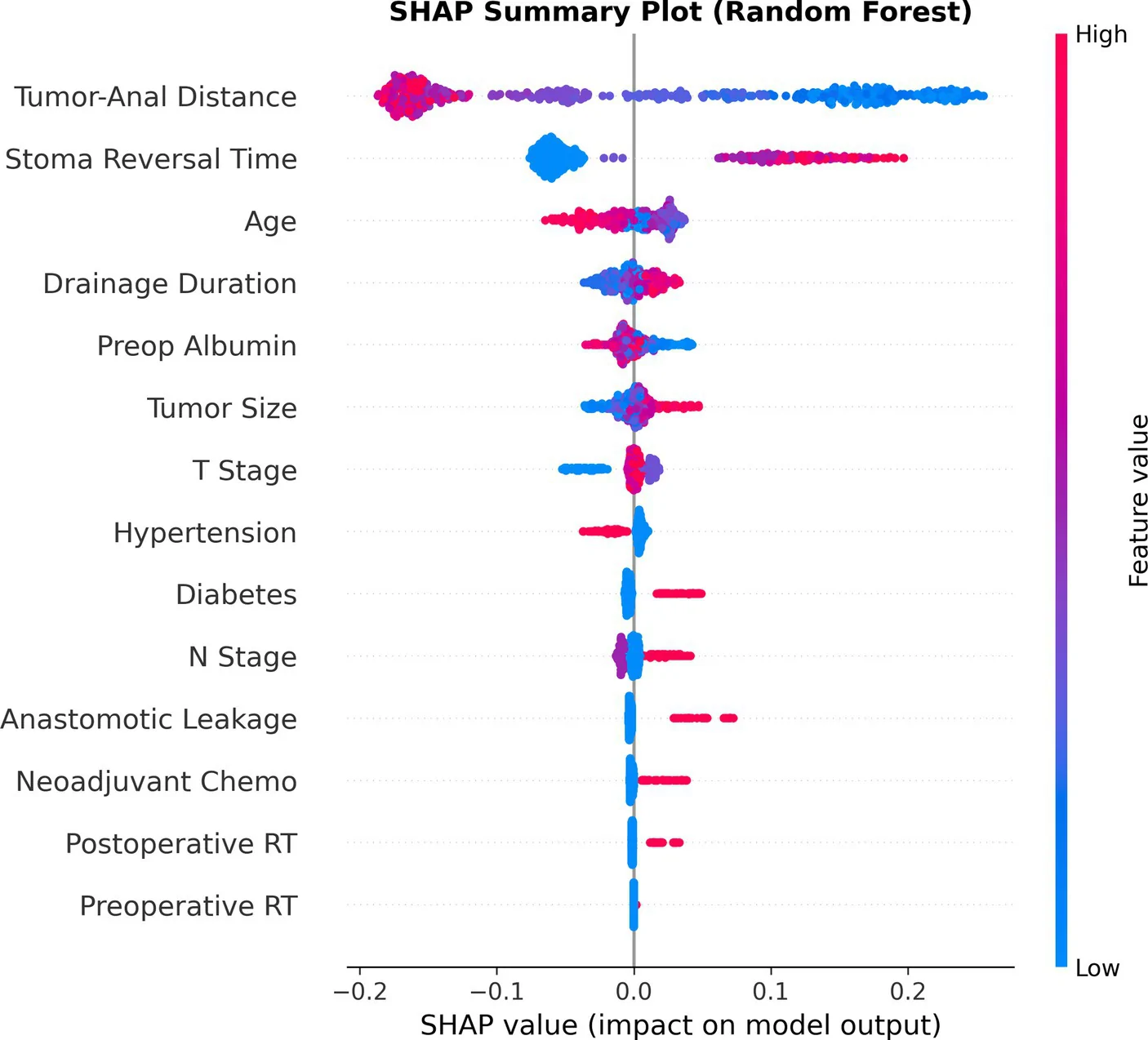
Forest plot of multivariate logistic regression odds ratios with 95% confidence intervals.

In external validation (Table 4; Figures 2–6), the RF model achieved the highest AUC of 0.903 (accuracy 0.835, sensitivity 0.871, specificity 0.796, F1 0.847), followed by the LR nomogram (AUC 0.888, accuracy 0.833). SVM (0.870), LightGBM (0.866), XGBoost (0.854), and MLP (0.840) ranked lower. XGBoost and LightGBM showed overfitting (training AUC ≥ 0.99), whereas RF and LR demonstrated consistent generalization.

**Table 4:** Predictive performance of six models in the training and external validation cohorts.

Model	Train AUC	Val AUC	Accuracy	Sensitivity	Specificity	F1	Brier
**Random Forest**	**0.890**	**0.903**	**0.835**	**0.871**	**0.796**	**0.847**	**0.150**
Logistic Regression	0.830	0.888	0.833	0.866	0.796	0.844	0.135
SVM	0.897	0.870	0.800	0.856	0.738	0.817	0.147
LightGBM	0.992	0.866	0.802	0.837	0.764	0.816	0.147
XGBoost	0.999	0.854	0.777	0.813	0.738	0.793	0.155
MLP	0.823	0.840	0.805	0.895	0.707	0.827	0.173

Accuracy through Brier score are for external validation. Best model (RF) in bold.

### 3.6 SHAP analysis

**Figure 7:**
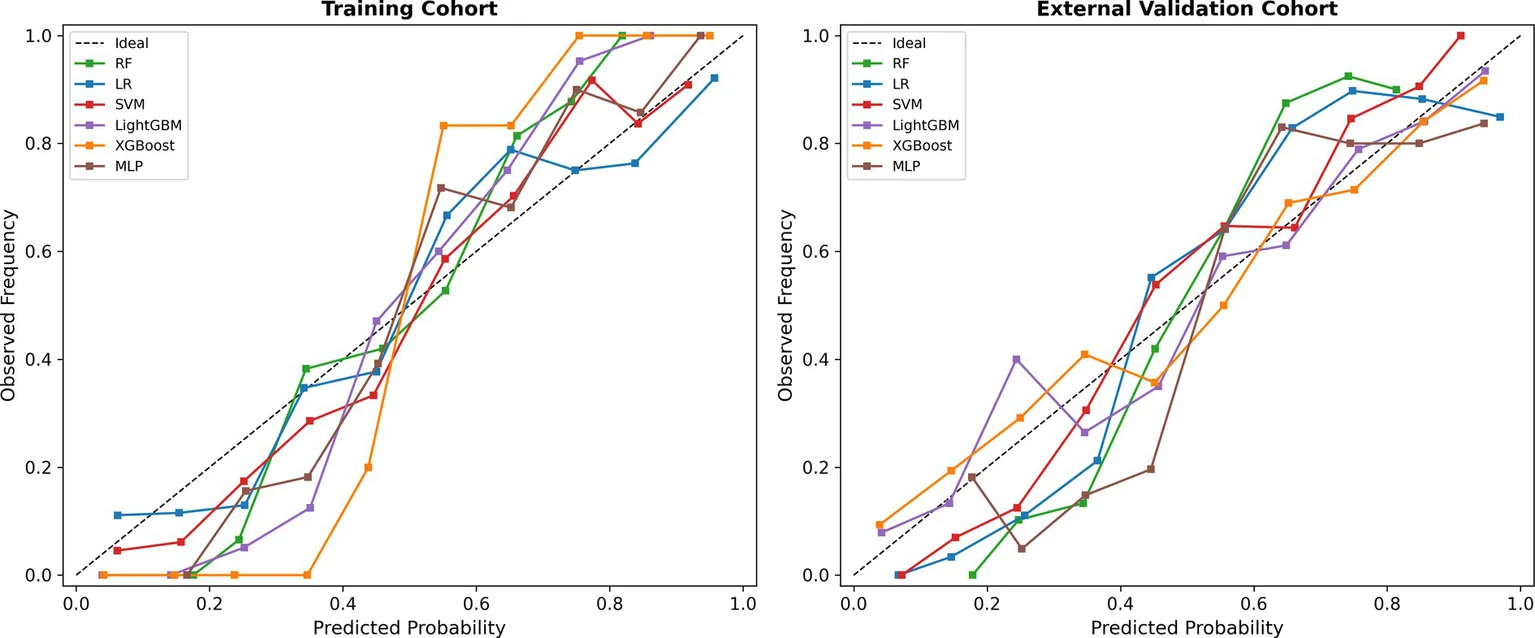
SHAP summary plot (beeswarm) of the random forest model.

**Figure 8:**
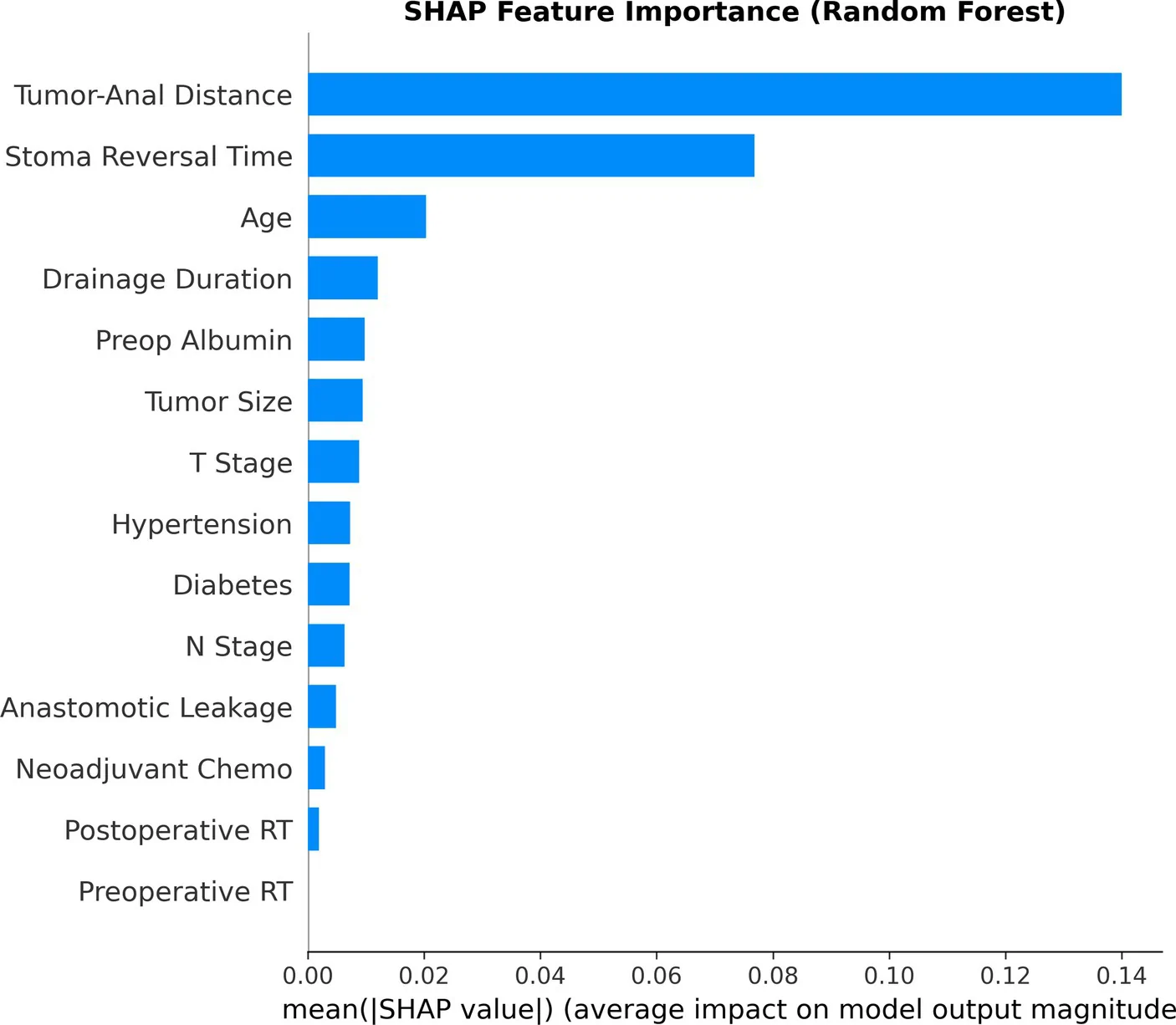
SHAP feature importance bar plot of the random forest model, ranked by mean absolute SHAP value.

SHAP analysis (Figures 7–8) revealed tumor–anal verge distance as the dominant predictor (mean |SHAP| = 0.140), followed by stoma reversal time (0.077), age (0.020), drainage duration (0.012), and preoperative albumin (0.010). Lower albumin values were consistently associated with higher predicted LARS risk, corroborating its role as a nutritional-inflammatory biomarker (18–21).

## 4 Discussion

In this multicenter retrospective cohort study of 906 patients from five hospitals, we developed and externally validated biomarker-integrated ML prediction models for major LARS following LAR for rectal cancer. Because several predictors, including stoma reversal time, anastomotic leakage, drainage duration, and postoperative radiotherapy, are available only after surgery or during follow-up, the full model should be positioned primarily as a postoperative or post-treatment risk-stratification tool. The RF model achieved the highest discriminative performance in external validation (AUC 0.903), slightly outperforming the conventional LR nomogram (AUC 0.888) and surpassing the external validation AUC of 0.852 reported by Qin et al. (22) in the largest previous multicenter ML study for LARS prediction. Both the RF model and nomogram demonstrated good calibration and clinical utility, providing actionable biomarker-driven risk stratification tools for personalized postoperative management.

The overwhelming dominance of tumor–anal verge distance as the primary predictor, confirmed by both multivariate regression (OR 0.733 per cm) and SHAP analysis (mean |SHAP| 0.140), is consistent with the well-established pathophysiological understanding that lower anastomoses result in greater disruption of the internal anal sphincter complex and reservoir function (4, 8, 17, 27). This finding corroborates prior reports identifying tumor height as the single most important determinant of LARS severity (5, 6, 15, 22). From a clinical perspective, this underscores the critical importance of careful preoperative assessment of tumor location when counseling patients about expected functional outcomes and considering the intensity of multimodal therapy.

The identification of anastomotic leakage as a potent independent risk factor (OR 7.027) has significant implications for both surgical practice and biomarker-guided postoperative surveillance. Anastomotic leakage triggers a cascade of pelvic inflammation, fibrosis, and scarring that permanently compromises neorectal compliance and sensory function (28, 41). This finding highlights the importance of leak prevention strategies—including meticulous surgical technique, adequate blood supply assessment, and appropriate use of diverting stomas in high-risk patients—as indirect means of LARS prevention (42). Diabetes mellitus also emerged as an independent risk factor (OR 2.793), consistent with its documented adverse effects on peripheral nerve function, microvascular integrity, and wound healing (43), all of which contribute to impaired anorectal physiology after pelvic surgery.

The role of treatment-related factors merits particular discussion in the context of biomarker-driven cancer therapy optimization. Neoadjuvant chemoradiotherapy, while critical for achieving pathological downstaging and improving local control in locally advanced rectal cancer, is increasingly recognized as a potent modulator of the tumor immune microenvironment (10–13). Recent neoadjuvant immune-response profiling studies have shown that treatment-related changes in T-cell subsets and the tumor immune microenvironment can be associated with therapeutic response (44), and immune-cell metabolic reprogramming is also increasingly recognized as a contributor to drug resistance and immune dysfunction (45). Radiation-induced immunogenic cell death, mucosal inflammation, and alterations in the pelvic nerve plexus have been implicated in long-term bowel dysfunction (15–17). In our study, preoperative radiotherapy was selected by LASSO as one of the strongest predictors, and neoadjuvant chemotherapy showed a borderline univariate association with LARS (*p* = 0.052). These findings align with Bregendahl et al. (15), who demonstrated significantly worse bowel function in irradiated vs. non-irradiated patients, and with Peeters et al. (16), who reported increased bowel dysfunction after short-course preoperative radiotherapy. The integration of these immunomodulatory treatment variables into our prediction framework reflects the growing recognition that treatment-related tissue injury—mediated in part through immune and inflammatory pathways—contributes meaningfully to post-treatment functional outcomes (10, 46). This observation is relevant to the ongoing paradigm shift toward organ-preserving, watch-and-wait, and de-escalation strategies in rectal cancer, where balancing oncological efficacy against long-term functional outcomes is a central consideration (13).

Preoperative serum albumin, included in our models as a nutritional-inflammatory biomarker, demonstrated an informative trajectory throughout the analytic pipeline. While it did not reach statistical significance in univariate (*p* = 0.956) or multivariate analysis (OR = 0.972, *p* = 0.370), it was retained by LASSO feature selection and ranked fifth in SHAP importance within the RF model, with lower values consistently associated with higher predicted LARS risk. This pattern suggests that albumin’s predictive contribution operates through nonlinear interactions with other variables that the parametric model cannot capture but the RF model can. Serum albumin reflects both nutritional status and systemic inflammatory burden (18, 20, 21); hypoalbuminemia has been associated with impaired tissue healing, compromised immune function, and adverse postoperative outcomes across surgical disciplines (19, 47, 48). The retention of albumin in the ML framework despite its non-significance in conventional regression illustrates a key advantage of ensemble methods in extracting predictive signal from clinically relevant but statistically marginal individual biomarkers (29, 30), supporting the biomarker integration paradigm central to personalized cancer therapy.

From a methodological standpoint, the comparison between ML models and the conventional nomogram yielded nuanced insights. While the RF model achieved the highest AUC, the LR nomogram performed remarkably well (AUC 0.888), demonstrating that a well-constructed parametric model can rival more complex algorithms for this particular clinical task, as also observed by Zhang et al. (24). Conversely, XGBoost and LightGBM showed clear evidence of overfitting, with near-perfect training AUCs (≥0.99) that degraded substantially upon external validation, highlighting the importance of rigorous multicenter external validation as emphasized by Steyerberg and Vergouwe (40). The RF model’s superior generalizability likely reflects the variance-reduction properties of bagging (35), particularly advantageous for heterogeneous multicenter data. Recent network-based colorectal cancer analyses (49) and explainable multi-omics biomarker discovery studies (50) further illustrate that computational models should be interpreted as decision-support or hypothesis-generating tools unless prospectively validated. The SHAP framework (31) provided valuable interpretability insights beyond traditional regression coefficients, revealing nonlinear dose–response relationships with direct clinical relevance.

This study has several limitations. First, the retrospective design introduces potential selection and information biases. Second, the full model is not a purely preoperative decision tool because several predictors are available only after surgery or during follow-up. Third, excluded-patient counts by specific reason could not be uniformly reconstructed across all centers, which may affect estimates of LARS incidence and model transportability. Fourth, although the validation cohort was multicenter, some participating hospitals contributed small sample sizes, particularly Ruijin General Hospital (*n* = 15), and center-level performance could not be robustly estimated. Fifth, our predictor set was limited to routinely available clinicopathological variables and did not include molecular biomarkers such as inflammatory cytokines, tumor-infiltrating lymphocyte counts, microbiome profiles, or immune checkpoint markers (11). Sixth, LARS was assessed at a single time point (6 months postoperatively) using self-reported scores (51), and anorectal manometry and ASA scores were not systematically available. Despite these limitations, the large overall sample size (*N* = 906), five-center design, strict TRIPOD adherence (32), comprehensive model comparison, and SHAP-based interpretability analysis strengthen the clinical relevance of our findings.

## 5 Conclusion

This multicenter study demonstrated that a biomarker-integrated random forest model incorporating 14 clinicopathological biomarkers—including nutritional indicators and treatment-related variables—achieved robust predictive performance for postoperative or post-treatment bowel dysfunction (major LARS), with an external validation AUC of 0.903. Tumor–anal verge distance, stoma reversal time, anastomotic leakage, and diabetes mellitus were identified as key independent risk factors. The random forest model modestly outperformed a conventional nomogram (AUC 0.888), while the nomogram remains a practical and interpretable alternative. These biomarker-driven prediction tools may facilitate postoperative risk stratification, shared decision-making regarding follow-up intensity, and targeted rehabilitation in rectal cancer survivors.

## Data Availability

The raw data supporting the conclusions of this article will be made available by the authors, without undue reservation.
